# Detection of autotrophic verrucomicrobial methanotrophs in a geothermal environment using stable isotope probing

**DOI:** 10.3389/fmicb.2012.00303

**Published:** 2012-08-17

**Authors:** Christine E. Sharp, Matthew B. Stott, Peter F. Dunfield

**Affiliations:** ^1^Department of Biological Sciences, University of CalgaryCalgary, AB, Canada; ^2^GNS Science, Extremophile Research UnitTaupo, New Zealand

**Keywords:** *Verrucomicrobia*, methane, methanotroph, stable isotope probing, “*Methylacidiphilum*”, acidophile

## Abstract

Genomic analysis of the methanotrophic verrucomicrobium “*Methylacidiphilum infernorum*” strain V4 has shown that most pathways conferring its methanotrophic lifestyle are similar to those found in proteobacterial methanotrophs. However, due to the large sequence divergence of its methane monooxygenase-encoding genes (*pmo*), “universal” *pmoA* polymerase chain reaction (PCR) primers do not target these bacteria. Unlike proteobacterial methanotrophs, “*Methylacidiphilum*” fixes carbon autotrophically, and uses methane only for energy generation. As a result, techniques used to detect methanotrophs in the environment such as ^13^CH_4_-stable isotope probing (SIP) and *pmoA*-targeted PCR do not detect verrucomicrobial methanotrophs, and they may have been overlooked in previous environmental studies. We developed a modified SIP technique to identify active methanotrophic *Verrucomicrobia* in the environment by labeling with ^13^CO_2_ and ^13^CH_4_, individually and in combination. Testing the protocol in “*M. infernorum*” strain V4 resulted in assimilation of ^13^CO_2_ but not ^13^CH_4_, verifying its autotrophic lifestyle. To specifically detect methanotrophs (as opposed to other autotrophs) via ^13^CO_2_-SIP, a quantitative PCR (qPCR) assay specific for verrucomicrobial-*pmoA* genes was developed and used in combination with SIP. Incubation of an acidic, high-temperature geothermal soil with ^13^CH_4_ + ^12^CO_2_ caused little shift in the density distribution of verrucomicrobial-*pmoA* genes relative to controls. However, labeling with ^13^CO_2_ in combination with ^12^CH_4_ or ^13^CH_4_ induced a strong shift in the distribution of verrucomicrobial-*pmoA* genes towards the heavy DNA fractions. The modified SIP technique demonstrated that the primary methanotrophs active in the soil were autotrophs and belonged to the *Verrucomicrobia*. This is the first demonstration of autotrophic, non-proteobacterial methanotrophy *in situ*, and provides a tool to detect verrucomicrobial methanotrophs in other ecosystems.

## Introduction

Methanotrophs are microorganisms that couple the oxidation of methane (CH_4_) to the reduction of sulfate, nitrate or molecular oxygen (Hanson and Hanson, 1996; Ettwig et al., 2009; Knittel and Boetius, 2009). The best studied of these are the aerobic methanotrophs. Until recently, all known species of methanotrophs belonged to the *Alphaproteobacteria* and *Gammaproteobacteria*. In 2007–2008, the first non-proteobacterial aerobic methanotrophs were isolated from geothermal areas in New Zealand, Italy and Russia (Dunfield et al., 2007; Pol et al., 2007; Islam et al., 2008). These methanotrophs form a distinct phylogenetic group within the *Verrucomicrobia* and have been given the proposed genus name “*Methylacidiphilum*” (Op den Camp et al., 2009). “*Methylacidiphilum*” strains are the most acidophilic methanotrophs known, capable of growing at <pH 1. They also display a thermophilic phenotype, with an upper growth temperature of 65°C (Op den Camp et al., 2009).

Aerobic methanotrophs convert methane to carbon dioxide (CO_2_) via a multistep pathway. In the first step, one atom of oxygen is incorporated into methane, generating methanol. There are two forms of the methane monooxygenase enzyme, a soluble form (sMMO) and a particulate form (pMMO). The pMMO is found in almost all methanotrophs isolated so far, while the sMMO has been found in a few. Three divergent *pmoCAB* operons encoding pMMO were detected in the genome of “*M. infernorum*” strain V4 but no genes encoding sMMO were found (Hou et al., 2008). Polymerase chain reaction (PCR) primers have previously been developed that are specific for the *pmoA* gene encoding the α-subunit of pMMO found in proteobacterial methanotrophs. Phylogenetic trees based on the 16S rRNA and *pmoA* genes are congruent (Kolb et al., 2003). This allows *pmoA* to be used as a target for the identification and quantification of methanotrophs in nature. However, the “universal” *pmoA* primers do not amplify “*Methylacidiphilum*” *pmoA* genes (Dunfield et al., 2007; Hou et al., 2008).

Formaldehyde is produced as a metabolic intermediate during the aerobic oxidation of methane. Proteobacterial methanotrophs assimilate formaldehyde via either the serine cycle to produce acetyl-CoA, or via the ribulose monophosphate (RuMP) pathway to produce glyceraldehyde-3-phosphate for biosynthesis. Analyses of the genome of “*M. infernorum*” strain V4 showed that key enzymes of both the RuMP pathway and serine cycle are absent (Hou et al., 2008). However, the genome does encode a ribulose-1,5-bisphosphate carboxylase/oxygenase (RuBisCo), and contains all other genes necessary to encode a complete Calvin-Benson-Bassham (CBB) cycle (Hou et al., 2008). This was surprising, as it was thought that carbon assimilation in methanotrophs via the CBB was unlikely due to the high ATP requirements of this pathway. Theoretically, using formaldehyde would be more efficient as this carbon is already in a partially reduced state. Recently, Khadem et al. (2011) showed that the closely related “*Methylacidiphilum fumarolicum*” strain SolV also encodes all the key enzymes of the CBB cycle. They verified experimentally using ^13^CO_2_ and ^13^CH_4_ that CO_2_ was its sole carbon source, while CH_4_ was used only for energy generation.

A common method for characterizing the active methanotrophic community in an environment is stable isotope probing (SIP) with ^13^CH_4_. In SIP, the labeled substrate is incorporated into cellular biomass, including DNA. The ^13^C-DNA can be resolved from ^12^C-DNA by density gradient ultracentrifugation. DNA isolated from the “heavy” or labeled fractions can be characterized taxonomically by 16S rRNA gene sequence analysis (Neufeld et al., 2007b), or for methanotrophs, via *pmoA* analysis (Cebron et al., 2007a,b; Neufeld et al., 2007a; Martineau et al., 2010; Redmond et al., 2010; Dumont et al., 2011). There are two fundamental problems with using SIP to detect verrucomicrobial methanotrophs: (i) because they are autotrophs they will not directly incorporate ^13^CH_4_; and (ii) mismatches in the primer binding regions mean that “universal” *pmoA* primers do not target their *pmoA* genes. These bacteria may have been overlooked in previous SIP studies. Therefore, we developed a modified SIP protocol based on labeling with ^13^CH_4_ and ^13^CO_2_ individually and in combination, and applied this method to identify active methanotrophic *Verrucomicrobia* in a geothermal soil. We also developed a quantitative PCR (qPCR) assay specific for verrucomicrobial-*pmoA* genes to use in combination with SIP gradient fractionation.

## Materials and methods

### Culture growth

“*M. infernorum*” strain V4 used in this study was originally isolated from the Hell's Gate geothermal area (Tikitere) in New Zealand. The composition and preparation of the growth medium were described previously (Dunfield et al., 2007). Stock cultures were maintained in 120-ml serum bottles containing 20 ml of culture medium, 0.25% (w/v) methanol and 10% CO_2_ (v/v) in the headspace.

### Soil collection

Soil samples were collected from the Hell's Gate geothermal area, Tikitere, in the spring of 2010. This site was where strain V4 was originally isolated. Soils were collected from various depths from the surface to 40 cm below the surface. Temperature was measured in the field using a handheld temperature probe model HI9060 (Hanna Instruments). The pH was measured upon return to the laboratory using an Accumet Basic AB15 pH meter (Fisher Scientific). Soils were immediately placed at 4°C and incubations were begun within 1 month of sampling.

### Soil methane oxidation

Five-gram (wet weight) amounts of soil from each sample depth (TIK3–9) were put into 120-ml serum bottles, which were sealed gas-tight with butyl rubber stoppers. Samples were set up in duplicate. 10% (v/v) CH_4_ and 10% (v/v) CO_2_ were added to the headspace and samples were incubated at near *in situ* temperatures (Table 1). Headspace CH_4_ mixing ratios were monitored using a Varian 450 gas chromatograph equipped with Hayesep N (0.5 m × 1/16″ × 1 mm) and Molsieve 13X (1.2 m × 1/16″ × 1 mm) columns in series (70°C), and a flame ionization detector (FID) (detector temperature 250°C). Mixing ratios were calculated by comparison with a known reference standard (Praxair). Methane oxidation rates were calculated using linear regression of mixing ratios in the first 5 days.

**Table 1:** **Physiochemical properties and methane oxidation rates of Tikitere soil profile samples TIK3–9**.

**Sample site**	**pH**	***In situ* temperature (°C)**	**Incubation temperature (°C)**	**Depth (cm)**	**Methane oxidation rate (μmol CH_4_ g^−1^ d^−1^)**
TIK3	2.7	36.8	37	1–5	0.7–1.3
TIK4	3.2	44.1	45	5–10	5.1–5.9
TIK5	3.5	58.2	55	10–15	6.2–7.0
TIK7	3.5	70.9	65	15–20	0.4–0.5
TIK8	3.9	81.6	75	20–30	ND
TIK9	2.0	90.2	75	30–40	ND

Data are based on duplicate measurements.

ND, not detected.

### SIP incubations

Strain V4 was inoculated into 40 ml of medium V42 (Dunfield et al., 2007) in 120 ml glass serum vials, in duplicate. 10% of ^12^CH_4_ or ^13^CH_4_ (99 atom% ^13^C) and 10% of ^12^CO_2_ or ^13^CO_2_ (99 atom% ^13^C) gases (Sigma-Aldrich) were added to the headspace in several combinations. Vials were incubated at 55°C on a rotary shaker at 120 rpm. Growth was measured via turbidity (OD600) using an Ultrospec 10 Cell Density Meter (Amersham Biosciences). For soil incubations, duplicate 5-g samples of soil from depths of 5–10 cm (TIK4) and 10–15 cm (TIK5), where the maximum CH_4_ oxidation rates were observed (Table 1), were added directly into 120 ml glass serum bottles. Vials were sealed and gases added as in the culture experiments. Duplicate samples of each treatment were incubated at 45 and 55°C, for TIK4 and TIK5 respectively. Headspace CH_4_ mixing ratios were monitored using gas chromatography for both soil and culture incubations. When more than 95% of the CH_4_ (14 days) was consumed, samples were frozen at −20°C for DNA extraction.

### DNA extraction, fractionation, and quantification

DNA was extracted from 0.5 g (±5 mg) of each soil using the FastDNA Extraction Kit for Soil (MP Biomedicals), with additional purification steps using 5.5 M guanidine thiocyanate (Knief et al., 2003). Unlabeled soil DNA from both TIK4 and TIK5 and genomic DNA from strain V4 were also prepared and used as controls to determine the expected position of unlabeled DNA in the cesium chloride (CsCl) density gradients. DNA centrifugation and fractionation were performed as described by Neufeld et al. (2007a,b). Briefly, ~500 ng of each DNA extract (^13^C-labeled and control) was combined with CsCl and gradient buffer into ultracentrifugation tubes. Ultracentrifugation was done at the maximum allowable speed of 50,000 rpm in the NVT90 rotor (Optima L-100K, Beckman Coulter Inc.) at 20°C with vacuum for 68 h. DNA was retrieved by gradient fractionation resulting in 12 fractions of approximately 400 μl each, where fraction 1 was the heaviest and fraction 12 was the lightest. The density of each fraction was measured with a refractometer (AR200, Reichert) to confirm gradient formation. DNA was precipitated from the CsCl with polyethylene glycol (PEG) and glycogen, washed with 70% ethanol and eluted in 30 ul of Tris-EDTA buffer. The DNA concentration of each fraction was determined with a Qubit Fluorometer using a Quant-iT™ dsDNA HS Assay Kit (Invitrogen).

### “*Methylacidiphilum” pmoA* primer design

“*Methylacidiphilum*”-specific *pmoA* primers were designed from a database of public-domain *pmoA* and *amoA* sequences (total 3131 sequences) using the ARB software package (Ludwig et al., 2004). Primers were designed to specifically target *pmoA* copies 1 and 2 from all three known strains of “*Methylacidiphilum*”. The following primers were designed for this study (5′→3′): V170f (GGA TWG ATT GGA AAG ATM G) and V613b (GCA AAR CTY CTC ATY GTW CC). The V170f and V613b primers had minimum of six mismatches to non-verrucomicrobial *pmoA* and *amoA* sequences in the database. PCR parameters were first optimized on a pure culture of strain V4 via gradient PCR (temperature range 48–58°C, MgCl_2_ range from 1.0 to 3.0 mM), optimal conditions are described in the section below. PCR products were purified with a QIAquick PCR Purification Kit (Qiagen) and cloned with the CloneJet PCR Cloning Kit (Fermentas) into JM109 Ca^2+^ competent cells. Cloned inserts were amplified from 20 colonies using the Clonejet pJET1.2 forward and reverse primers (Fermentas). Positive PCR products were purified with a QIAquick PCR Purification Kit (Qiagen) and screened via Restriction Fragment Length Polymorphism (RFLP) using the HhaI restriction enzyme. Two restriction digest patterns were observed and duplicates of each were sequenced to verify their identity. Sequenced inserts displayed 100% identity to *pmoA*1 and *pmoA*2 genes from strain V4. Verrucomicrobial-*pmoA* primers produced no positive PCR amplicons when tested on genomic DNA extracts of *Methylosinus trichosporium* OB3b, *Methylocystis parvus* OBBp and *Methylobacter luteus* (NCIMB 11914); and DNA extracts of soil methanotroph enrichment cultures containing strains related to *Crenothrix*, *Methylocystis*, *Methylococcus*, and *Methylobacter* (data not shown).

### Real-time PCR quantification

PCR assays were prepared using a QIAgility (v4.13.5) instrument (Qiagen). Briefly, 1 μl of DNA was added to 11.5 μl of Master mix containing: 6.25 μl of 2 × RotorGene SYBR Green PCR Master Mix (Qiagen), 1 μM of each primer V170f and V613b and 4.75 μl of RNase free water (Qiagen). Each measurement was performed in duplicate. qPCR was performed on the RotorGene Q and respective software (Qiagen). Assays were performed with a three-step thermoprofile: an initial denaturation of 5 min at 94°C; 40 cycles of 94°C for 60 s, 56°C for 45 s and 72°C for 45 s; and a final elongation step of 72°C for 10 min. Fluorescence data acquisition occurred during the last step of each cycle. The specificity of each reaction was verified by melt curve analysis, in addition to running selective PCR products on an agarose gel to verify their size. Serial dilutions of PCR-amplified *pmoA* from strain V4 were used as calibration standards for the real-time assays. The positive control *pmoA* PCR extracts were purified using an EZ-10 Spin Column PCR Purification Kit (BioBasic Inc.) as per the manufacturer's instructions. The purified PCR product obtained was then quantified via a Qubit Fluorometer using a Quant-iT™ dsDNA HS Assay Kit (Invitrogen). Measured DNA concentration was then converted to target molecules per microliter and *pmoA* standards were adjusted to 10^8^, 10^6^, 10^4^, and 10^2^ target molecules μl^−1^ for storage at −20°C. Efficiencies for standard curves used to calculate qPCR values ranged from 86–94%.

### 16s rRNA gene pyrotag sequencing

Microbial communities associated with the labeled and unlabeled fractions as identified by verrucomicrobial-*pmoA* qPCR were investigated using 16S rRNA gene pyrotag sequencing. 16S rRNA genes were amplified from the gradient fractions using FLX Titanium amplicon primers 454T_RA_X and 454T_F containing the 16S rRNA gene targeted primers 926f and 1392r at their 3′- ends, along with adaptors necessary for the Roche Titanium chemistry (Ramos-Padron et al., 2011). Each reverse primer was tagged with a unique 10-nucleotide identifier barcode sequence allowing sequences to be separated according to sample. PCR mixtures contained 0.04 μM of the forward primer, 25 μl of 2 × Premix F (Interscience), 1.25 U *Taq* DNA polymerase (Fermentas), 0.04 μM of the reverse primer (with its unique barcode sequence for each sample) and 2 μl of template DNA, made up to 50 μl total with nuclease-free water (Qiagen). PCR reaction conditions were: initial denaturation at 95°C for 3 min, followed by 35 cycles of 30 s at 95°C, 45 s at 55°C and 90 s at 72°C, and a 10-min final elongation at 72°C. PCR products were visualized on a 1% agarose gel and purified with an EZ-10 Spin Column PCR Purification Kit (BioBasic Inc.). DNA concentration was determined via a Qubit Fluorometer using a Quant-iT™ dsDNA HS Assay Kit (Invitrogen). Purified PCR products (typically 150 ng total DNA) were analysed at the Genome Quebec and McGill University Innovation Centre, Montreal, Quebec with a Genome Sequencer FLX Instrument, using a GS FLX Titanium Series Kit XLR70 (Roche Diagnostics Corporation). Sixty samples were multiplexed on a single run; in total 6379–9710 reads were obtained per sample. The QIIME software platform (Caporaso et al., 2010) was used to analyze the sequences. QIIME removes low-quality sequences from the set based on a user-defined threshold (a minimum quality score of 25 was selected), clusters Operational Taxonomic Units (OTU) based on 97% identity, identifies chimeric sequences via ChimeraSlayer (Haas et al., 2011) and classifies the sequences via BLAST (Altschul et al., 1990) based on the Greengenes database for bacterial and archaeal 16S rRNA gene amplicons (Desantis et al., 2006).

### Nucleotide sequence accession numbers

Representative 16S rRNA gene sequences obtained in this study from have been deposited in the GenBank/EMBL/DDBJ databases under accession numbers JX141439-JX141447.

## Results

### Soil characteristics

Results of the soil analyses are shown in Table 1. All soils from the depth profile had acidic pH values in the range of 2.0–3.9. Temperatures ranged from 36.8°C at the surface to 90.2°C at the deepest point sampled. At a depth below 20 cm the soils were mostly volcanic ash containing chunks of precipitated elemental sulfur.

### Methane oxidation rates

Methane oxidation was detected in the soil to a depth of 20 cm (TIK7). No methane oxidation was detected compared to a negative control below a depth of 20 cm, corresponding to incubation temperatures of >65°C (Table 1). The methane oxidation rates of samples TIK4 (5–10 cm) and TIK5 (10–15 cm) were higher than all other soils, at 5.1–5.9 and 6.2–7.0 μmol CH_4_ g wet weight^−1^ day^−1^, respectively. For this reason, samples TIK4 and TIK5 were chosen for DNA-SIP incubations.

### ^13^C-labeling of DNA in strain V4

**Figure 1:**
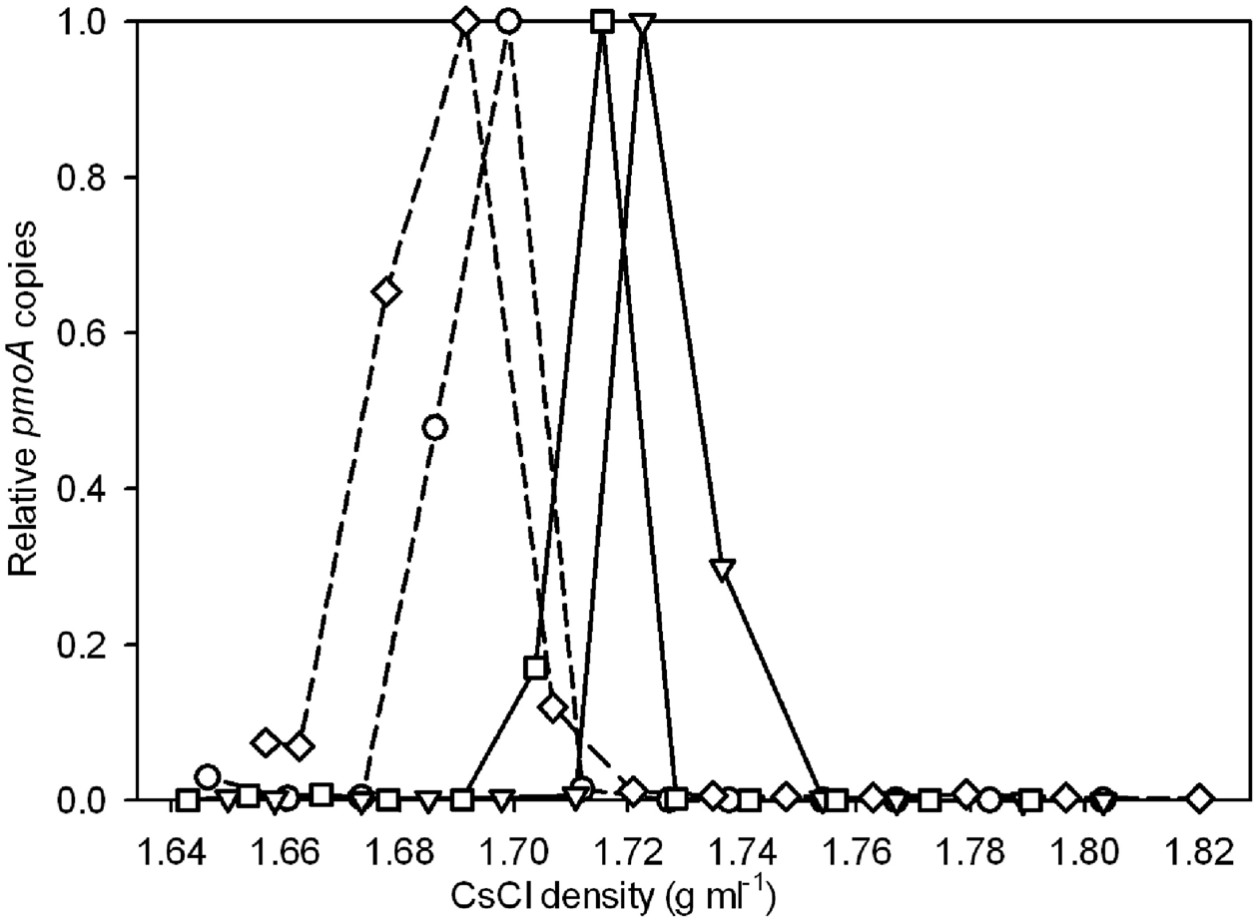
**Relative *pmoA* gene abundances recovered from CsCl gradient fractions for strain V4 after incubation with ^13^CH_4_ + ^12^CO_2_ (○), ^13^CH_4_ + ^13^CO_2_ (∇), ^12^CH_4_ + ^13^CO_2_ (□), and a ^12^CH_4_ + ^12^CO_2_ control (⋄).** Solid lines represent incubations with ^13^CO_2_ and dashed lines represent incubations with ^12^CO_2_. The y-axis indicates the relative abundance of *pmoA* genes recovered from each gradient fraction, with the highest quantity detected in any gradient fraction set to 1.0. Relative abundances rather than absolute *pmoA* gene counts are used to facilitate comparison across the different experiments. Points are averages of duplicate qPCR measurements. Experiments were performed in duplicate, one representative experiment is shown as duplicate experiments showed similar results.

The incorporation of ^13^C into DNA of strain V4 was measured by quantification of *pmoA* gene copies in the individual SIP fractions. Strain V4 grown in the presence of ^13^CH_4_ + ^12^CO_2_ showed little or no shift in density of the DNA compared to unlabeled DNA (Figure 1). Incubation with either ^13^CH_4_ + ^13^CO_2_ or ^12^CH_4_ + ^13^CO_2_ showed an increase in DNA density as compared to the unlabeled DNA, indicating that the substrate required for ^13^C-labeling was ^13^CO_2_ (Figure 1). No growth was observed in cultures of strain V4 incubated in the presence of ^13^CO_2_ without added CH_4_, as determined by OD600.

### SIP of geothermal soil

**Figure 2:**
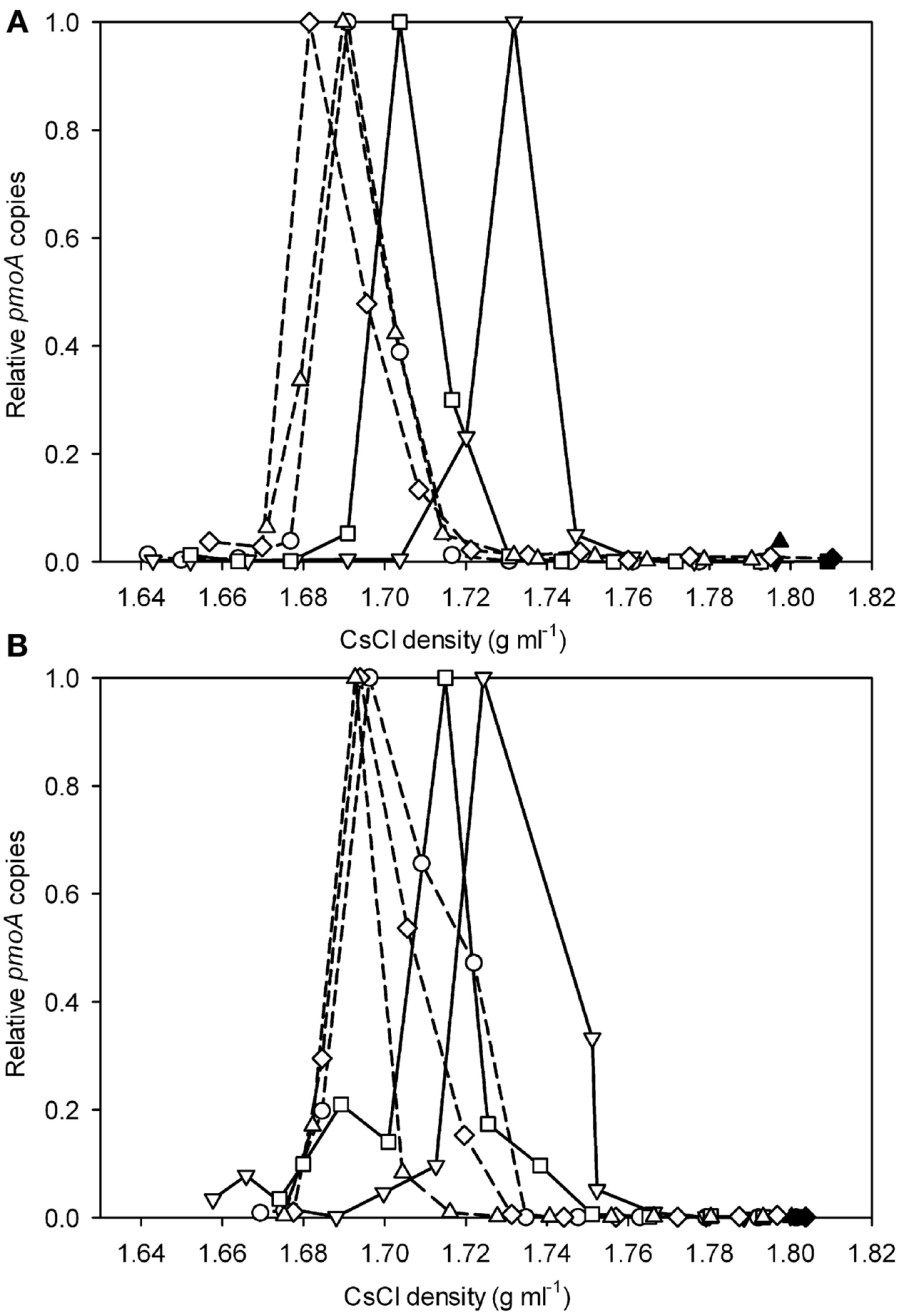
**Relative *pmoA* gene abundances recovered from CsCl gradient fractions for (A) TIK4 and (B) TIK5 after incubation with ^13^CH_4_ + ^12^CO_2_ (○), ^13^CH_4_ + ^13^CO_2_ (∇), ^12^CH_4_ + ^13^CO_2_ (□), ^13^CO_2_ (⋄), and a control (△).** Solid lines represent incubations with ^13^CO_2_ and dashed lines represent incubations with ^12^CO_2_. The y-axis indicates the relative abundance of *pmoA* genes recovered from each gradient fraction, with the highest quantity detected from a gradient fraction equal to 1.0. Points are averages of duplicate qPCR measurements. Experiments were performed in duplicate, one representative graph is shown as duplicate experiments showed similar results.

Applying the verrucomicrobial-*pmoA* qPCR system to TIK4 (Figure 2A) and TIK5 (Figure 2B) soils showed that labeling with ^13^CH_4_ + ^12^CO_2_ or ^13^CO_2_ + no CH_4_ resulted in little or no change in the density distribution of the *pmoA* genes relative to the unlabeled soil control. Labeling with ^13^CH_4_ + ^13^CO_2_ or ^12^CH_4_ + ^13^CO_2_ showed a clear shift in the distribution of *pmoA* genes towards the heavy fractions relative to the unlabeled soil control (Figures 2A,B).

**Figure 3:**
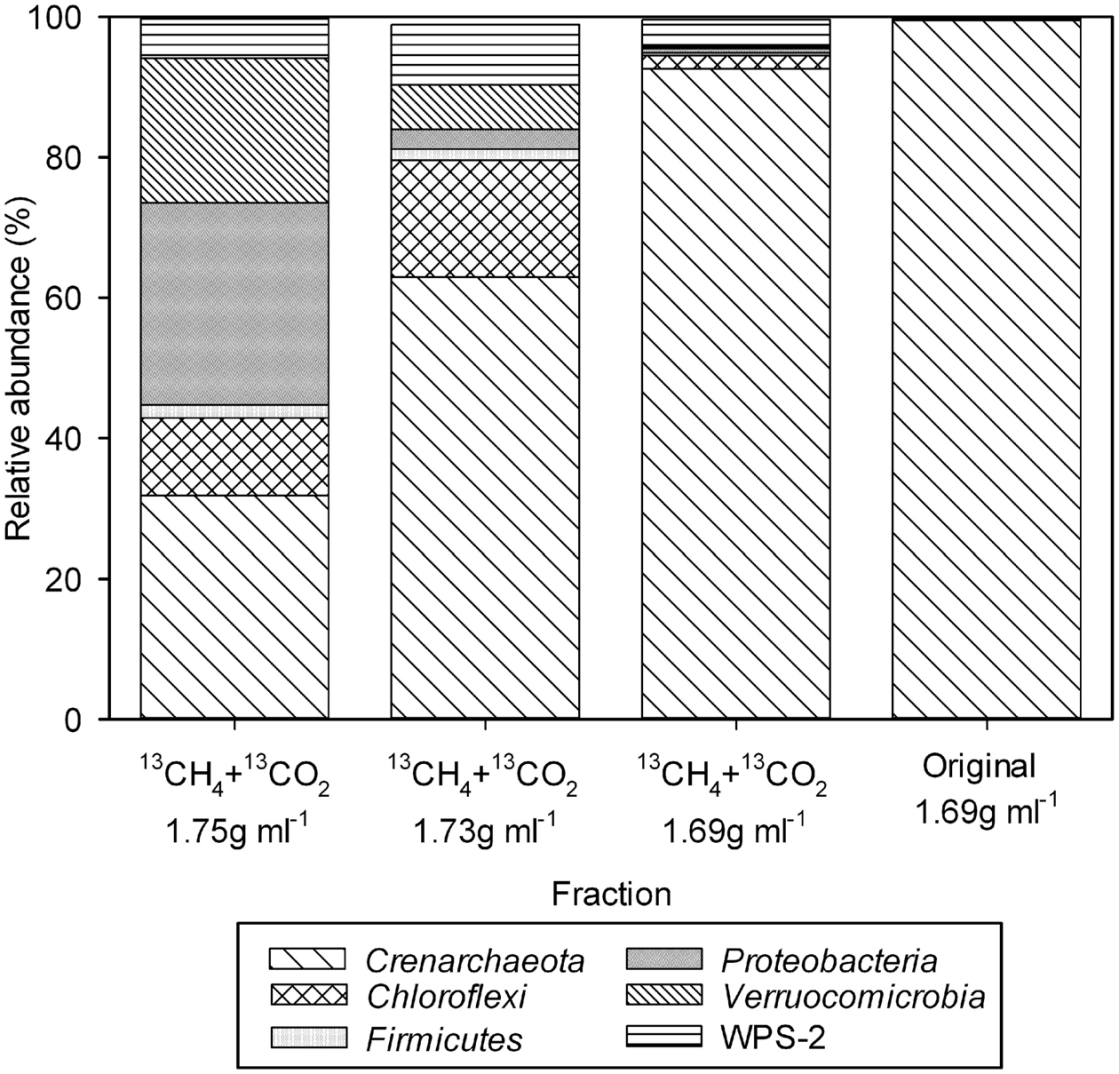
**Relative abundance of different bacterial and archaeal phyla from heavy DNA (density 1.73 g ml^−1^ and 1.75 g ml^−1^) and light DNA (density 1.69 g ml^−1^) from the TIK5 ^13^CH_4_ + ^13^CO_2_ SIP experiment, and the control soil (density 1.69 g ml^−1^).** 16S rRNA gene sequences were clustered based on 97% identity and classified via BLAST (Altschul et al., 1990) based on the Greengenes database of bacterial and archaeal 16S rRNA gene amplicons (Desantis et al., 2006).

To identify the microorganisms associated with the heavy and light fractions as identified by qPCR, 16S rRNA gene pyrotag sequencing was performed. From the TIK5 incubation with ^13^CH_4_ + ^13^CO_2_ the two heaviest fractions with the highest relative abundances of *pmoA* genes (at densities of 1.75 g ml^−1^ and 1.73 g ml^−1^) and a light fraction at a density of 1.69 g ml^−1^ were chosen for analysis (Figure 2B). The fraction with the highest abundance of *pmoA* genes (at a density of 1.69 g ml^−1^) in the DNA extracted directly from control TIK5 soil (Figure 2B) was also sequenced. 16S rRNA genes could not be amplified from the control DNA fractions at densities of 1.73 and 1.75 g ml^−1^, indicating that in SIP experiments only 13-C labeled DNA populated these fractions. No major differences in community composition were observed between the “light” DNA fraction from the ^13^CH_4_ + ^13^CO_2_ labeling experiment and the control soil DNA fraction (Figure 3). Members of the *Crenarchaeota* were found in relative abundances greater than 90% in both fractions. “*Methylacidiphilum*” was found in low abundance in both these fractions at less than 0.5% of total reads.

However, a distinct change in the microbial community was observed between the heavy and light fractions (Figure 3). Both of the heavy fractions from TIK5 ^13^CH_4_ + ^13^CO_2_ enrichment showed an increase in the abundance of the *Verrucomicrobia* relative to the light fractions. “*Methylacidiphilum*” was found at ~6% of all sequence reads at a density of 1.73 g ml^−1^ and ~20% of all sequence reads at a density of 1.75 g ml^−1^ Members of the phyla *Crenarchaeota, Chloroflexi, Firmicutes, Proteobacteria*, and candidate division WPS-2 were also found in high abundance in the heavy fractions. As the bacteria detected from these phyla were not suspected to be methanotrophs, their presence in the heavy DNA could reflect a large amount of ^13^CO_2_ labeling of other lithoautotrophs using sulfur, ammonium, or other energy sources at this geothermal site, or it could indicate cross-feeding on products of methane oxidation.

**Figure 4:**
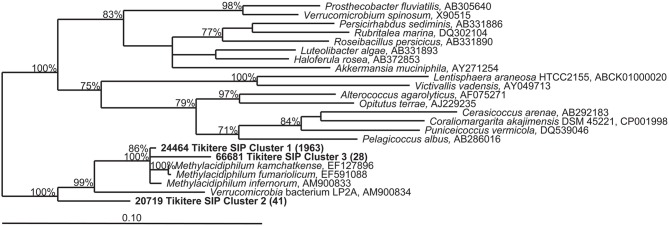
**Phylogenetic tree of partial (423 nucleotides) 16S rRNA gene sequences belonging to the *Verrucomicrobia* detected in the TIK5 soil sample.** Representative sequences of each cluster were obtained from 16S rRNA pyrotag data using QIIME (Caporaso et al., 2010). Clusters included primarily sequences detected in the heavy DNA fractions of the ^13^CH_4_ + ^13^CO_2_ SIP experiment. The tree was constructed with TREE PUZZLE, a quartet maximum-likelihood method, using a Schoeniger-von Hasseler distance calculation (Schmidt et al., 2002) and 10,000 iterations. Support values greater than 70% for the major nodes are given. The scale bar represents 0.1 changes per nucleotide position. Bracketed numbers indicate the number of sequences detected per cluster (from 33,702 total reads).

QIIME was used to simultaneously categorize all samples and create multi-sample OTUs (or clusters). The dominant “*Methylacidiphilum*” 16S rRNA gene cluster showed 99.6% similarity via BLAST (Altschul et al., 1990) to the 16S rRNA gene of strain V4. The three most abundant 16S rRNA gene clusters (>25 sequences, >400 nucleotides in length, and found in more than one extract) identified as *Verrucomicrobia* by QIIME were 99.6, 96.2, and 91.6% identical to strain V4, respectively (Figure 4). The cluster most closely related to strain V4 (Cluster 1) was the most predominant with 1963 sequence reads. Although clusters 1 and 3 contained sequences from both the heavy fractions and light fractions, cluster 2 only contained sequences from the heavy fractions (data not shown). However, all clusters were more predominant in the heavy DNA than the light.

## Discussion

Our experiments with “*M. infernorum*” strain V4 indicated that it was autotrophic, confirming the work of Khadem et al. (2011) on the closely related bacterium “*M. fumarolicum*” strain SolV. Khadem et al. (2011) present several lines of evidence suggesting that strain SolV is autotrophic. All the genes encoding enzymes necessary for the CBB cycle are expressed, with the gene encoding the RuBisCO being the most prominent. SDS-PAGE gels identified RuBisCO as one of the most dominant proteins in the cell extracts of strain SolV. Finally, the ^13^C label percentage in the biomass experiments agreed with the ^13^C label percentage of CO_2_ in the cultures confirming that biomass carbon is exclusively derived from CO_2_. In our study, labeling a pure culture of strain V4 with ^13^CH_4_ in a background of 10% ^12^CO_2_ produced little shift in the density of the DNA, indicating that methane is not a major carbon source for this bacterium. However, DNA from strain V4 showed a clear density shift when labeled with ^13^CH_4_ + ^13^CO_2_ or ^12^CH_4_ + ^13^CO_2_ as compared to DNA from a culture grown in the presence of ^13^CH_4_ + ^12^CO_2_ or ^12^CH_4_ + ^12^CO_2_. This confirmed that CO_2_ is the major carbon source for strain V4. Labeling with ^13^CH_4_ + ^13^CO_2_ resulted in a greater density shift towards the heavy fractions as compared to labeling with ^12^CH_4_ + ^13^CO_2_ likely due to the dilution of the ^13^CO_2_ pool by ^12^CO_2_ produced from the oxidation of ^12^CH_4_.

We initially tried SIP experiments on the Tikitere soil using ^13^CH_4_ in the absence of added CO_2_. Despite high soil methane oxidation rates, these experiments displayed no detectable enrichment of verrucomicrobia in the heavy DNA fractions (data not shown), and thereby provided the impetus for the more in-depth studies presented here. Although in theory addition of ^13^CH_4_ alone should label verrucomicrobial methanotrophs via the ^13^CO_2_ produced from ^13^CH_4_ oxidation, in practice the extent of labeling was very small. We assume that there was sufficient natural ^12^CO_2_ in the soil to dilute any labeling via the ^13^CO_2_ produced from ^13^CH_4_. These verrucomicrobia are naturally light (40.8–45.5% GC; Op den Camp et al., 2009) and probably a lot of labeling is required to bring them out of the broad peak of community DNA in a CsCl gradient. With the sensitive qPCR assay we did see a minor shift in *pmoA* density when using ^13^CH_4_ (+^12^CO_2_) in the soil SIPs, but this shift was much smaller than that observed when using ^13^CO_2_. Labeling with ^13^CH_4_ alone is therefore possible, but only weakly enriches for heavy DNA of verrucomicrobial methanotrophs when there is a natural or experimentally enriched background of ^12^CO_2_. When methane SIP experiments are performed, ^12^CO_2_ is usually added to prevent cross-labeling of other autotrophs with produced ^13^CO_2_ (Neufeld et al., 2007a,b). We hypothesized that previous SIP experiments would overlook verrucomicrobia, and that a proper SIP experiment would instead require ^13^CO_2_. Labeling with ^13^CO_2_ will also target other autotrophs, but as the target methanotrophs are themselves autotrophs, there is essentially no way around this problem, and no advantage of using ^13^CH_4_ alone.

When labeling the soils with ^13^CH_4_ and ^13^CO_2_ individually and in combination it was not possible to use the relative amounts of DNA in each density fraction to determine the extent of labeling. Total DNA profiles did not necessarily demonstrate methanotroph enrichment, because incubation with ^13^CO_2_ also resulted in labeling of the autotrophic community in the geothermal soils. Additionally, most methane SIP experiments have observed some ^13^C uptake by organisms utilizing byproducts of the oxidation pathway, such as methanol (Hutchens et al., 2004; Cebron et al., 2007b; Redmond et al., 2010). To specifically detect “*Methylacidiphilum*” in ^13^CO_2_-SIP a qPCR assay specific for the verrucomicrobial-*pmoA* genes was developed. Applying this technique to geothermal soils from the Tikitere geothermal area, NZ, revealed a clear shift of the *pmoA* counts towards the heavy fractions when labeling with ^13^CH_4_ + ^13^CO_2_ or ^12^CH_4_ + ^13^CO_2_ in both samples. Labeling with ^13^CH_4_ + ^13^CO_2_ produced a heavier *pmoA* peak (density ~1.73 g ml^−1^) than the heavy *pmoA* peak (density ~1.71 g ml^−1^) generated by labeling with ^12^CH_4_ and ^13^CO_2_ (Figures 2A,B). This is likely due to the generation of unlabeled ^12^CO_2_ from the oxidation of ^12^CH_4_ by the methanotrophic community, followed by assimilation of some of this ^12^CO_2_. The density range of the heavy and light peaks from the soil SIP incubations correlate with the corresponding peaks from the experiment using strain V4, providing verification of our results.

A distinct change in the microbial community was observed between the light and heavy fractions (Figure 3). Although the light fraction from TIK5 ^13^CH_4_ + ^13^CO_2_ and the original extract were dominated by members of the *Crenarchaeota* phylum, the heavy fractions from TIK5 ^13^CH_4_ + ^13^CO_2_ showed a more diverse microbial community of *Verrucomicrobia, Crenarchaeota, Chloroflexi, Firmicutes, Proteobacteria*, and candidate division WPS-2 (Figure 3). All *Verrucomicrobia* clusters identified by QIIME were related to the genus “*Methylacidiphilum*” with a maximum sequence divergence of 8.4%. Phylogenetic analysis of the representative *Verrucomicrobia* cluster sequences showed three distinct clades (Figure 4). The first cluster (Cluster 1) contained the majority of methanotrophic *Verrucomicrobia* sequences obtained from 16S rRNA gene pyrotag sequencing and was nearly identical (99.6%) to “*M. infernorum*” strain V4. Cluster 3 was 96.2% similar to strain V4. The presence of a more divergent clade (Cluster 2) is interesting, as it suggests that there may be considerable evolutionary diversity within the methanotrophic *Verrucomicrobia*. This cluster was only found in the heavy SIP fractions, strongly suggesting that it is autotrophic. Based on its relationship to “*Methylacidiphilum*”, it likely also represents a methanotroph, although one not particularly abundant in the original soil sample. The pyrosequencing procedure is notoriously error-prone, however each of the representative sequences shown in Figure 4 was carefully inspected for chimeric sections manually and by ChimeraSlayer (Haas et al., 2011), and for potential homopolymer errors. Each cluster was detected in multiple samples, and the 8.4% maximum divergence is greater than the expected 454 sequencing error of about 1.07% (Gilles et al., 2011).

No proteobacterial methanotrophs could be detected in any SIP gradient fraction from Tikitere geothermal soils using standard *pmoA* primers 189 and 682 or 189 and 661 (data not shown) (McDonald et al., 2008). Proteobacterial methanotrophs were detected in very low abundance in the light fractions of the 16S rRNA gene pyrotag datasets (<0.03% of total reads), verifying these are not the primary active methanotrophs in this environment. The only potential proteobacterial methanotroph strain detected was related to *Methylocella*.

Geothermal soil samples examined in this study had the capacity to oxidize methane up to 65°C (Table 1). This is consistent with the highest growth temperature recorded for “*Methylacidiphilum*” (Op den Camp et al., 2009). Proteobacterial methanotrophs have been found with a higher growth temperature, for example *Methylothermus* sp. strain HB has an upper growth temperature of 72°C (Bodrossy et al., 1999). However, no proteobacterial methanotroph has been found in an environment as acidic as Tikitere (Dunfield, 2009). Soil methane oxidation rates were highest at a depth of 15 cm, with a maximum potential rate of 7.0 μmol CH_4_ g wet weight^−1^ d^−1^ at 55°C. A 2003 study by Knief et al. (2003) found that methane oxidation rates in upland soils ranged from <0.01 to 74.6 nmol CH_4_ g^−1^ d^−1^ with most soils below 24.0 nmol CH_4_ g^−1^ d^−1^. Methane oxidation rates in wetlands such as peat bogs and rice paddies usually range from 0.17 to 80 μmol CH_4_ g^−1^ d^−1^ (Kravchenko, 2002; Wagner et al., 2005; Kip et al., 2010; Graef et al., 2011; Lee et al., 2011; Barbier et al., 2012). The rates in the methane rich geothermal soil are therefore similar to rates measured in methane-rich wetlands rather than upland soils.

The dominance of *Crenarchaeota* in both the heavy SIP fractions and the control sample suggests that they dominate the geothermal system at Tikitere. Some members of the *Crenarchaeota* phylum have previously been shown to be autotrophic ammonia and sulfur oxidizers that fix CO_2_ (Hallam et al., 2006; Nicol and Schleper, 2006; Marakushev and Belonogova, 2010; Benson et al., 2011). It is not unexpected that they dominate this site due to the high levels of ammonia (e.g., 420 mmol mol^−1^) and hydrogen sulfide, H_2_S (e.g., 84 mmol mol^−1^) present (Giggenbach, 1994). A recent study by Benson et al. (2011) found that *Crenarchaeota* lineages dominated the archaeal community in steam vents and caves in Hawaii Volcanoes National Park, Yellowstone National Park and Lassen Volcanic National Park. Interestingly, some of the *Crenarchaeota* grouped phylogenetically with the ammonia-oxidizing *Crenarchaeota*. BLAST analysis of representative *Crenarchaeota* sequences from Tikitere showed low sequence similarity (less than 87%) to any cultured representative (data not shown), indicating these are novel lineages of *Crenarchaeota*. The dominant *Chloroflexi* sequence detected showed 99% sequence similarity to *Chloroflexi* bacterium T104, and *Proteobacteria* representatives showed 94–96% sequence similarity to *Acidicaldus* sp. T163. Both of these bacteria were previously cultivated from the same soil (Stott et al., 2008). Little is known regarding candidate division WPS-2 as it is rarely detected in soils, however, it previously has been identified in a cold fumarole on Socompa Volcano, Puna de Atacama, Andes (Costello et al., 2009).

The analysis of genomic data from strain V4 (Hou et al., 2008) was the key to informing the ecological studies described in this paper. It allowed the development of a verrucomicrobial-*pmoA* qPCR detection system to specifically detect the organism in SIP experiments. The genome analysis also indicated the potential for purely autotrophic methanotrophy, a process confirmed experimentally by Khadem et al. (2011). The autotrophic nature of “*Methylacidiphilum*” makes its detection via ^13^CH_4_-SIP problematic when there is a natural or experimentally enriched background of ^12^CO_2_ to dilute the pool of ^13^CO_2_ produced from ^13^CH_4_ oxidation. We therefore used a combination of ^13^CH_4_ + ^13^CO_2_ to achieve optimal labeling. The drawback of the method is the difficulty in distinguishing autotrophic methanotrophs from other autotrophs in the system, so the technique will be less useful for discovery of novel methanotrophic groups than for studying and expanding a known methanotrophic group like the “*Methylacidiphilum*”. The use of functional genes like *pmoA* provides specific information on methanotrophs, although our ability to design broad-target primers is still limited by the small size of the database. A ^13^CO_2_ SIP control experiment without methane will also be a partial control to describe the community of other autotrophs. This study describes the first detection of an active verrucomicrobial methanotroph community in any environment using SIP, and provides *in situ* verification of the autotrophic nature of the methanotrophic *Verrucomicrobia*. We are currently applying this technique to other environments to detect verrucomicrobial methanotrophs that may have been overlooked in previous studies.

### Conflict of interest statement

The authors declare that the research was conducted in the absence of any commercial or financial relationships that could be construed as a potential conflict of interest.
